# Statistical approach to optimize production of biosurfactant by *Pseudomonas aeruginosa* 2297

**DOI:** 10.1007/s13205-014-0203-3

**Published:** 2014-03-08

**Authors:** Arthala Praveen Kumar, Avilala Janardhan, Seela Radha, Buddolla Viswanath, Golla Narasimha

**Affiliations:** Applied Microbiology Laboratory, Department of Virology, Sri Venkateswara University, Tirupati, 517 502 India

**Keywords:** *Pseudomonas aeruginosa*, Biosurfactant, Kinetic growth modeling, Plackett–Burman, Response surface methodology (RSM)

## Abstract

The main objective of this paper is to optimize biosurfactant production by *Pseudomonas aeruginosa* 2297 with statistical approaches. Biosurfactant production from *P. aeruginosa* 2297 was carried out with different carbon sources, and maximum yield was achieved with sawdust followed by groundnut husk and glycerol. The produced biosurfactant has showed active emulsification and surface-active properties. From the kinetic growth modeling, the specific growth rate was calculated on sawdust and it was 1.12 day^−1^. The maximum estimated value of product yield on biomass growth (*Y*_p/*x*_) was 1.02 g/g. The important medium components identified by the Plackett–Burman method were sawdust and glycerol along with culture parameter pH. Box–Behnken response surface methodology was applied to optimize biosurfactant production. The obtained experimental result concludes that Box–Behnken designs are very effective statistical tools to improve biosurfactant production. These results may be useful to develop a high efficient production process of biosurfactant. In addition, this type of kinetic modeling approach may constitute a useful tool to design and scaling-up of bioreactors for the production of biosurfactant.

**Electronic supplementary material:**

The online version of this article (doi:10.1007/s13205-014-0203-3) contains supplementary material, which is available to authorized users.

## Introduction

Microbial surfactants are structurally different group of surface-active biomolecules produced by a variety of microorganisms and are receiving considerable attention due to their unique properties such as higher biodegradability, lower toxicity, and greater stability (Mukherjee et al. 2006; Mulligan 2005). Biosurfactants are predominantly produced by bacteria, fungi, and yeasts include glycolipids, lipoaminoacids, lipopeptides, lipoproteins, lipopolysaccharides, phospholipids, monoglycerides, and diglycerides. Among these, the glycolipids produced by strains of *Pseudomonas* have received much attention due to their notable tensioactive and emulsifying properties (Maier and Soberon-Chavez 2000; Mulligan 2005). However, biosurfactants have limited applications owing to their high production costs, which can be lowered by process optimization, downstream processing strategies, agro-industrial waste fermentation, and use of hyper-producer strains (e.g., mutant and recombinant strains) (Wei et al. 2004; Perfumo et al. 2010). One of the methods which accomplished the above objective is the selection of suitable media components and optimal culture conditions to enhance biosurfactant productivity. The limitations of classical method of media optimization can be overcome by the application of single factor optimization process by statistical experimental design using Plackett–Burman design and response surface methodology (RSM) (Lotfy et al. 2007; Tanyildizi et al. 2005).

The Plackett–Burman design is a widely used statistical design technique for the screening of the medium components, and the variables screened by Plackett–Burman design were further optimized in a 2^3^ factorial Box–Behnken design methodology (Plackett and Burman 1944; Box 1952). Response surface methodology (RSM) is the extensively used statistical technique for media optimization and for designing experiments, evaluating the effects of factor and relative significance and searching the optimum factors related to desired response. It has the intense ability to interpret the interactive effects among input variables are some attractive features of RSM (Al-Araji et al. 2007; Montgomery 1997). In the present study, we have applied response surface methodology (RSM) to enhance the production of biosurfactant by *Pseudomonas aeruginosa* 2297.

## Materials and methods

### Test organism

*Pseudomonas aeruginosa* (MTCC 2297) was obtained from the microbial type culture collection (MTCC), Chandigarh, India, and it was maintained on nutrient slants at 4 °C.

### Examination of biosurfactant produced by *Pseudomonas aeruginosa*

#### Oil spreading method and oil collapse method

Oil spreading technique and oil collapsed method were carried out according to the method (Youssef et al. 2004).

### Optimization of carbon and renewable sources for enhanced production of biosurfactant

*P*. *aeruginosa* was cultivated in 250 ml Erlenmeyer flasks containing 50 ml mineral salt medium (MSM) (g/l) (Camilios et al. 2008). To this medium, different carbon sources (2 %), i.e., glycerol, glucose, coconut oil, and groundnut oil, and different renewable sources (10 %), i.e., rice bran, sawdust, groundnut husk, and wheat bran, were added and sterilized at 121 °C, 15 lbs pressure for 15 min. After sterilization, the flasks were cooled to room temperature, and then, 2 % of overnight culture was inoculated and incubated at 30 °C in an orbital shaker for 6 days. For every 24 h, the broth was collected to analyze growth and biosurfactant production and their emulsification and surface activities.

### Experimental design and statistical analysis

#### Plackett–Burman design

To find out the important medium components, a Plackett–Burman design was applied and it is a design of fractional plan. It allows the investigation of up to *N* − 1 variables with *N* experiments and assumes that there are no interactions between the different media components. For this study, six components were selected to evaluate their effect on biosurfactant production in 12 experiments, and surface tension was used as a response. Each column represents a different experimental trial, and each row represents different variables. Each variable was tested at two levels, a higher (+) and a lower (–) level (Table 1).

**Table 1:** High and low levels of factors with coded settings by PBD

Trial	*X*_1_	*X*_2_	*X*_3_	*X*_4_	*X*_5_	*X*_6_	Surface tension (mN/m)
	Sawdust (gm)	Groundnut husk (g)	Glycerol (ml)	Groundnut oil (ml)	pH	Inoculum level (ml)	
R1	+1 (10)	+1 (10)	+1 (3)	+1 (3)	+1 (9)	+1 (5)	41.32
R2	−1 (5)	+1 (10)	−1 (1)	+1 (3)	+1 (9)	+1 (5)	39.11
R3	−1 (5)	−1 (5)	+1 (3)	−1 (1)	+1 (9)	+1 (5)	40.02
R4	+1 (10)	−1 (5)	−1 (1)	+1 (3)	−1 (5)	+1 (5)	69.31
R5	−1 (5)	+1 (10)	−1 (1)	−1 (1)	+1 (9)	−1 (1)	49.03
R6	−1 (5)	−1 (5)	+1 (3)	−1 (1)	−1 (5)	+1 (5)	68.23
R7	−1 (5)	−1 (5)	−1 (1)	+1 (3)	−1 (5)	−1 (1)	70.62
R8	+1 (10)	−1 (5)	−1 (1)	−1 (1)	+1 (9)	−1 (1)	43.24
R9	+1 (10)	+1 (10)	−1 (1)	−1 (1)	−1 (5)	+1 (5)	69.04
R10	+1 (10)	+1 (10)	+1 (3)	−1 (1)	−1 (5)	−1 (1)	68.51
R11	−1 (5)	+1 (10)	+1 (3)	+1 (3)	−1 (5)	−1 (1)	68.72
R12	+1 (10)	−1 (5)	+1 (3)	+1 (3)	+1 (9)	−1 (1)	41.33

R1–R12 represents twelve different fermentations

### Response surface methodology

The optimized concentrations and interactions between the significant factors were identified by Plackett–Burman design and studied by using response surface methodology. RSM was used for experimental design based on the Box–Behnken design algorithm. The factors settings were tabulated (Table 3). MATLAB version 7.7.0 (R2008b) was used to create a 3-factor Box–Behnken design. The generalized polynomial model of three factors was as follows:

1 $$Y = \beta_{0} + \, \beta_{1} X_{1} + \beta_{2} X_{2} + \beta_{3} X_{3} + \, \beta_{12} X_{1} X_{2} + \, \beta_{13} X_{1} X_{3} + \, \beta_{23} X_{2} X_{3} + \, \beta_{11} X_{1}^{2} + \beta_{22} X_{2}^{2} + \, \beta_{33} X_{3}^{2}$$

where *Y*––predicted response of fermentation *X*_1_, *X*_2_ and *X*_3_ are the coded settings for three factors *β*_0_––value of fitted response at the center point of the design *β*_1_, *β*_2_, and *β*_3_––linear coefficients *β*_12_, *β*_13_, and *β*_23_––interaction coefficients *β*_11_, *β*_22_, and *β*_33_––quadratic coefficients.

### Emulsification index (*E*_24_)

The emulsifying capacity was evaluated by an emulsification index (*E*_24_). The *E*_24_ of culture samples was determined by adding 2 ml of oil and 2 ml of the cell-free broth to a test tube, vortexed at high speed for 2 min, and allowed to stand for 24 h. The *E*_24_ index is given as percentage of the height of emulsified layer (cm) divided by the total height of the liquid column (cm). The percentage of emulsification index was calculated by using the following equation (Tabatabaee et al. 2005; Sarubbo et al. 2006).

$$E_{ 2 4} = {\text{Height of emulsion formed}} \times 100/{\text{total height of solution}}.$$

### Extraction of biosurfactant

Initially, culture supernatant was adjusted to pH of 2.0 by adding 5 mol/l H_2_SO_4_ for precipitation of biosurfactant. The precipitates were extracted with two volumes of ethyl acetate. After vacuum evaporation of the solvents using rotary evaporator, crude biosurfactant was extracted with pellet form. The yielded pellets were applied onto thin-layer chromatography (TLC). Solvent mixture used in this study was chloroform, methanol, and water (65:25:4, V/V/V). Biosurfactant spots were detected by using orcinol reagent (Zhang Guo-liang et al. 2005).

### Quantification of biosurfactant by orcinol method

The culture supernatant is acidified to pH 2 with 5 mol/l H_2_SO_4_ and kept at 4 °C overnight. Two hundred microliters of acidified culture supernatant was extracted three times with 1 ml of diethyl ether. Then, fractions were pooled, dried, and resuspended in 1 ml of 0.05 M sodium bicarbonate. Two hundred microliters of sample was treated with 1.8 ml of a orcinol solution (100 mg orcinol in 53 % H_2_SO_4_ and boiled for 20 min). After cooling at room temperature for 15 min, the readings are taken at 421 nm. Biosurfactant concentration was calculated from standard curves prepared with l-rhamnose and expressed as rhamnose equivalents (in mg/ml) (Chandrasekaran and BeMiller 1980; Koch et al. 1991).

### Determination of surface tension

For surface tension measurements, 5 ml of broth supernatant was transferred to a glass tube that was submerged in a water bath at a constant temperature (28 °C). Surface tension was calculated by measuring the height reached by the liquid when freely ascended through a capillary tube (Munguia and Smith 2001). As control, non-inoculated broth was used, and the surface tension was calculated according to the following formula:

$$\gamma = \frac{rh\delta g}{2}$$

*γ* = surface tension (mN/m); *d* = density (g/ml); *g* = gravity (980 cm/s^2^); *r* = capillary radius (0.05 cm); *h* = height of the liquid column (cm).

### Bacterial growth and dry cell weight

Cell growth was determined by monitoring the optical density of culture broth at 600 nm. The biomass was determined from the cells after centrifugation of the culture broth at 10,000 rpm (6,700*g*) 4 °C for 10 min. The dry cell weight (DCW) was obtained from the cell pellets by washing twice with distilled water and drying in hot air oven at 105 °C for 24 h (Suwansukho et al. 2008).

### Kinetic model for growth and glycolipid product

Logistic equation: The sigmoidal shape of batch growth of *Pseudomonas aeruginosa* can be given as

2 $$\frac{{{\text{d}}X}}{{{\text{d}}t}} = \mu X\left( {1 - \frac{X}{{X_{\infty } }}} \right)$$

where *μ*: specific growth rate, *X*_∞_: maximum biomass concentration.

Logistic equations are a set of equations that characterize growth in terms of specific growth rate (Shuler and Kargi 2003). The integration of Eq. (2) with the boundary condition *X*(0) = *X*_0_ yields the logistic curve.

3 $$X = \frac{{X_{0} {\text{e}}^{\mu t} }}{{1 - \frac{{X_{0} }}{{X_{\infty } }}\left( {1 - {\text{e}}^{\mu t} } \right)}}$$

The above equation can be written as follows:

4 $$\mu = {{\frac{1}{{\overline{X} }}\frac{\varDelta X}{\varDelta t}} \mathord{\left/ {\vphantom {{\frac{1}{{\overline{X} }}\frac{\varDelta X}{\varDelta t}} {\left( {1 - \frac{{\overline{X} }}{{X_{\infty } }}} \right)}}} \right. \kern-0pt} {\left( {1 - \frac{{\overline{X} }}{{X_{\infty } }}} \right)}}$$

$\overline{X}$: Average biomass concentration.

Leudeking–Piret model: Bio-products are classified as growth associated, non-growth associated, and mixed growth associated metabolites based on their production during log or stationary phases of cell growth. The kinetics of rate of product formation (*r*_p_), in batch culture was described by Leudeking–Piret model and was given as follows:

5 $$r_{\text{p}} = \frac{dp}{dt} = \alpha \left( {r_{x} } \right) + \beta x$$

where α––growth associated term; β––non-growth associated term.

The bio-product formation associated with exponential growth of *Pseudomonas aeruginosa* was modeled as

6 $$r_{\text{p}} = \frac{dp}{dt} = \alpha \left( {r_{x} } \right)$$

A mixed growth associated product formation was modeled by the Eq. (8), and the product produced during stationary phase was modeled as follows:

7 $$r_{\text{p}} = \frac{{{\text{d}}p}}{{{\text{d}}t}} = \beta x$$

The above equation is rearranged to calculate *α* and *β* by plotting a graph between *r*_p/*x*_ and *r*_*x*_/*x*.

The product yield on biomass growth,

8 $$Y_{P/X} = \frac{{X - X_{0} }}{{P - P_{0} }}.$$

## Results

**Fig. 1:**
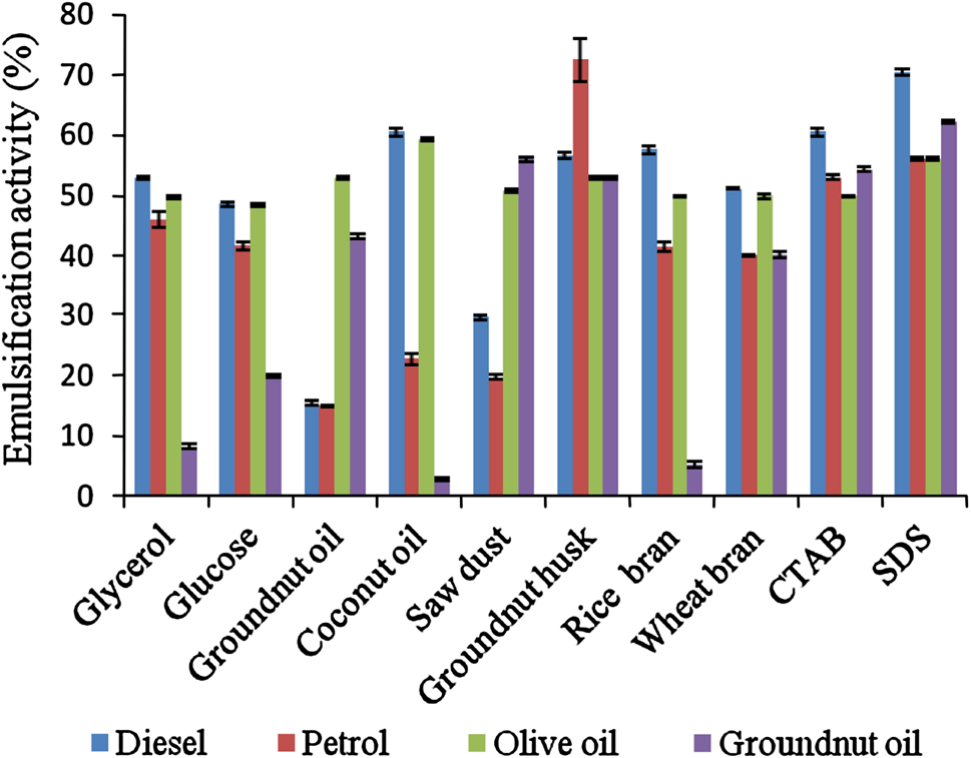
Emulsification activity of different carbon and renewable sources from *P. aeruginosa*. *Values are represented as mean ±SD

**Fig. 2:**
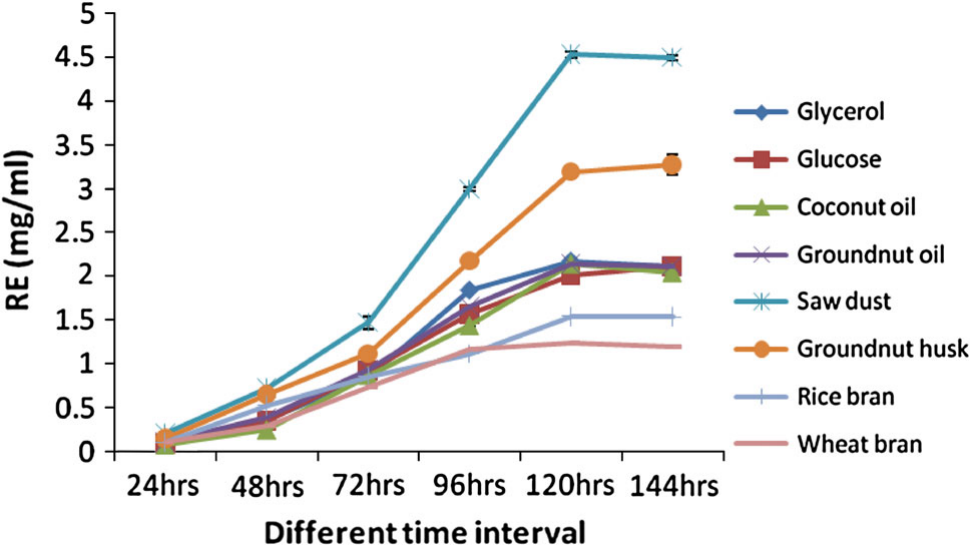
Rhamnose equivalents of different carbon and renewable sources from *P. aeruginosa*. *Values are represented as mean ±SD

In the present study, the screening of *Pseudomonas aeruginosa* 2297 was carried out using oil collapse and oil spreading techniques. It gave positive results for both oil collapse and oil spreading tests. The emulsification activities of the biosurfactant produced by *Pseudomonas aeruginosa* 2297 from different fermentation substrates and synthetic surfactants were tested with diesel, petrol, olive oil, and groundnut oil. When petrol and olive oil tested, maximum emulsification activity of 72.25 ± 2.47 and 59.23 ± 0.19 % was shown by biosurfactant produced from groundnut husk and coconut oil, respectively, used as a substrate and was comparable to all synthetic surfactants (Fig. 1). In diesel and groundnut oil, the synthetic surfactants (SDS) had the highest activity of 70.41 ± 0.56 and 62.31 ± 0.28 %, respectively, compared to all biosurfactants (Fig. 1). From the quantification experiment, the highest biosurfactant production was observed in sawdust with 4.53 ± 0.03 mg/ml (Fig. 2) followed by groundnut husk and glycerol as substrates.

**Fig. 3:**
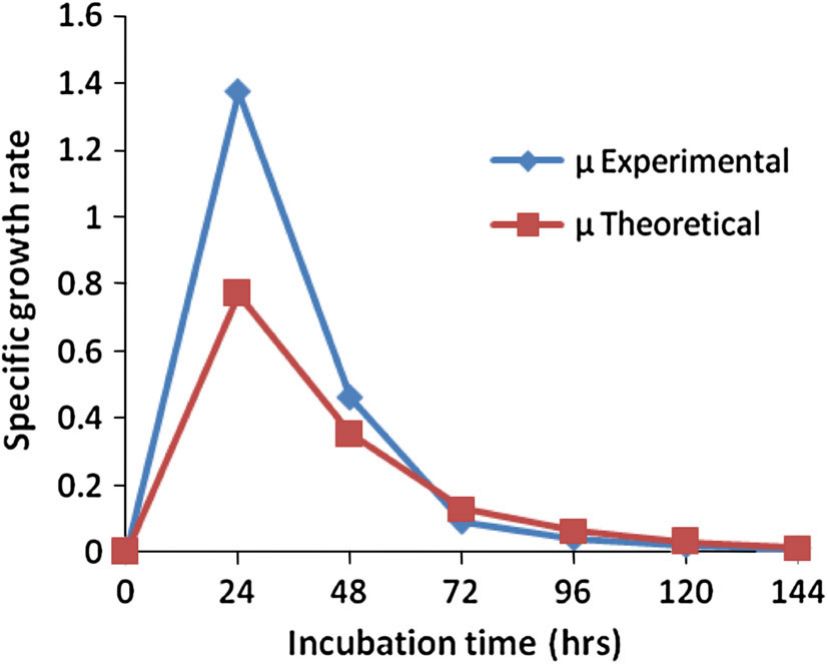
Comparison of experimental and predicted specific growth rate of *P. aeruginosa*

The biomass concentration on sawdust was used in the calculation of specific growth rate, and it was obtained as 1.12 day^−1^, which would describe the most of the data. The lower value of specific growth rate (0.04 h^−1^) was observed on cheap substrate (sawdust). The variation in experimental and predicted specific growth rate with time was depicted in Fig. 3. Higher value of *F*(11.14) and a very low value of *P* (<0.01) indicate that the logistic equation was the best-fit growth model for the *Pseudomonas aeruginosa* 2297 growth on sawdust consumption (Supplementary Table 1).

**Fig. 4:**
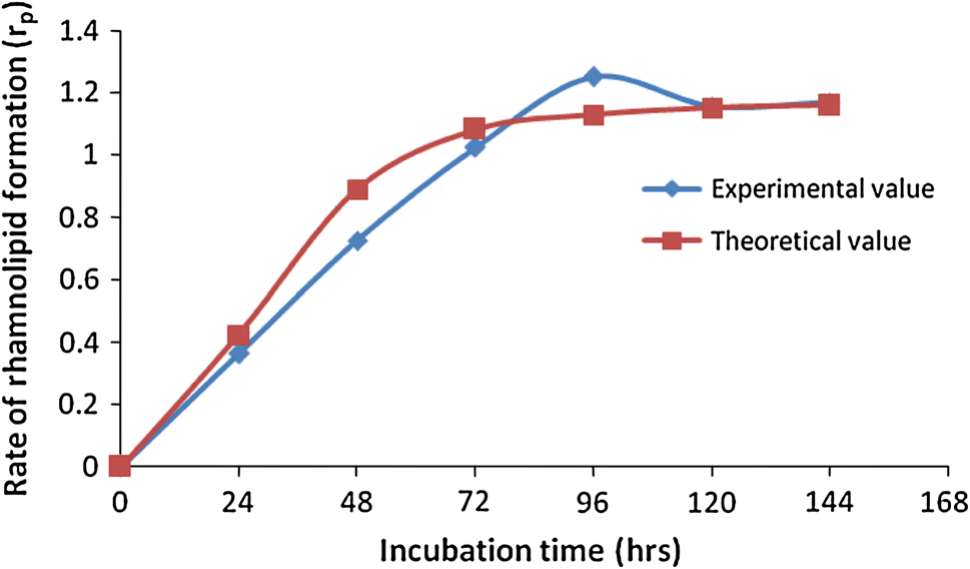
Comparison of experimental and predicted rate of rhamnolipid formation (*r*_*p*_) of *P. aeruginosa*

Based on the data obtained on the biomass and biosurfactant concentrations, the growth rate (*r*_*x*_) and product formation (*r*_p_) were calculated. The value of *β* was obtained as 0.186 with the negligible *α* which was an indication of the bio-product was non-growth associated product. From the above results, it was noticed that the more amount of product was formed during the stationary phase of *Pseudomonas aeruginosa* 2297. The comparison of experimental and predicted rate of biosurfactant production was depicted (Fig. 4). The estimated value of product yield on biomass growth (*Y*_p/*x*_) was 1.02 g/g. The significance of model was verified with analysis of variance given in Supplementary Table 2. The higher value of *F*(13.19) and a very low value of *P* (<0.001) indicated the best fit of model to the experimental data.

**Fig. 5:**
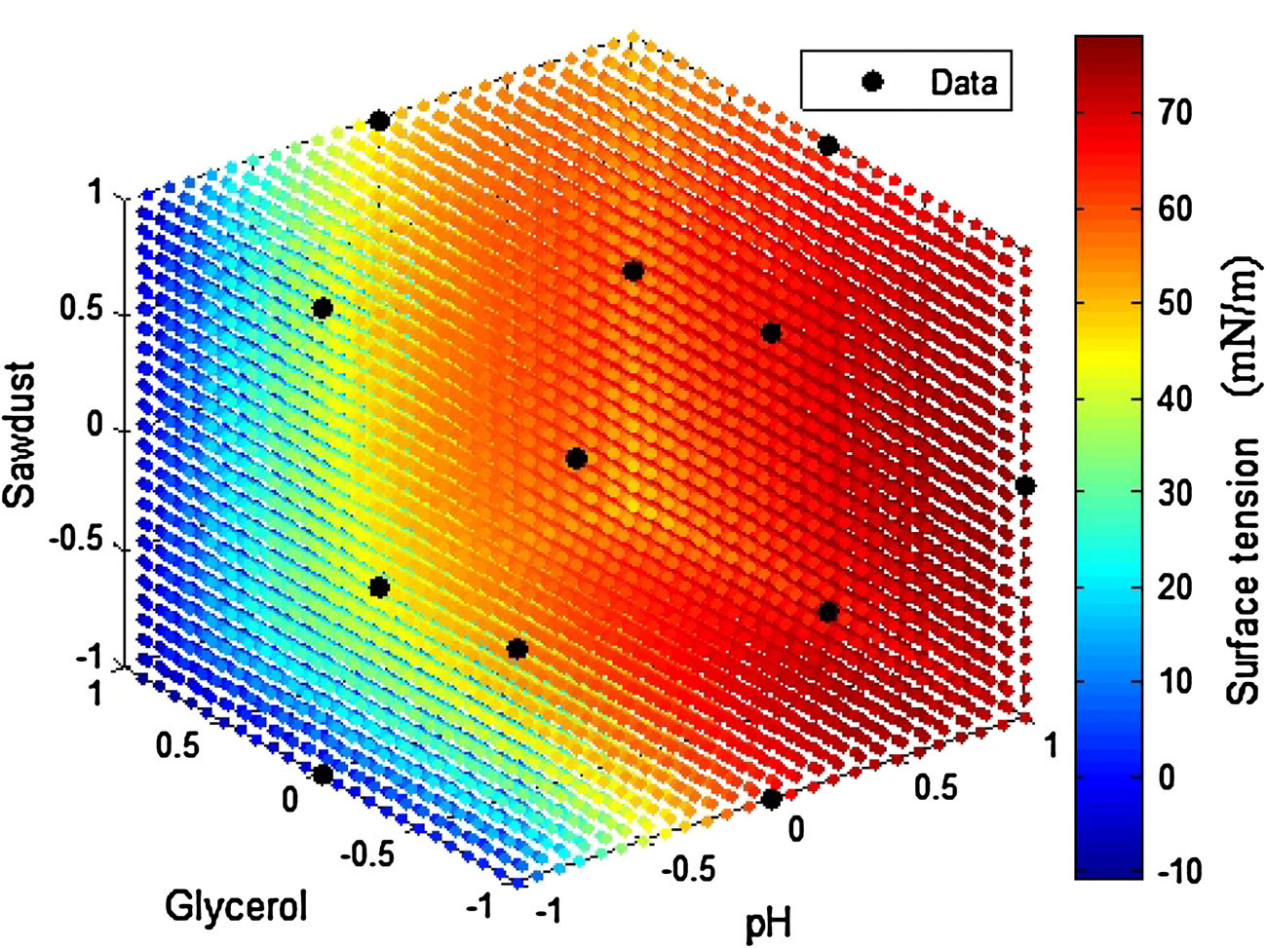
Quadratic surface model for biosurfactant production by *P. aeruginosa*

**Fig. 6:**
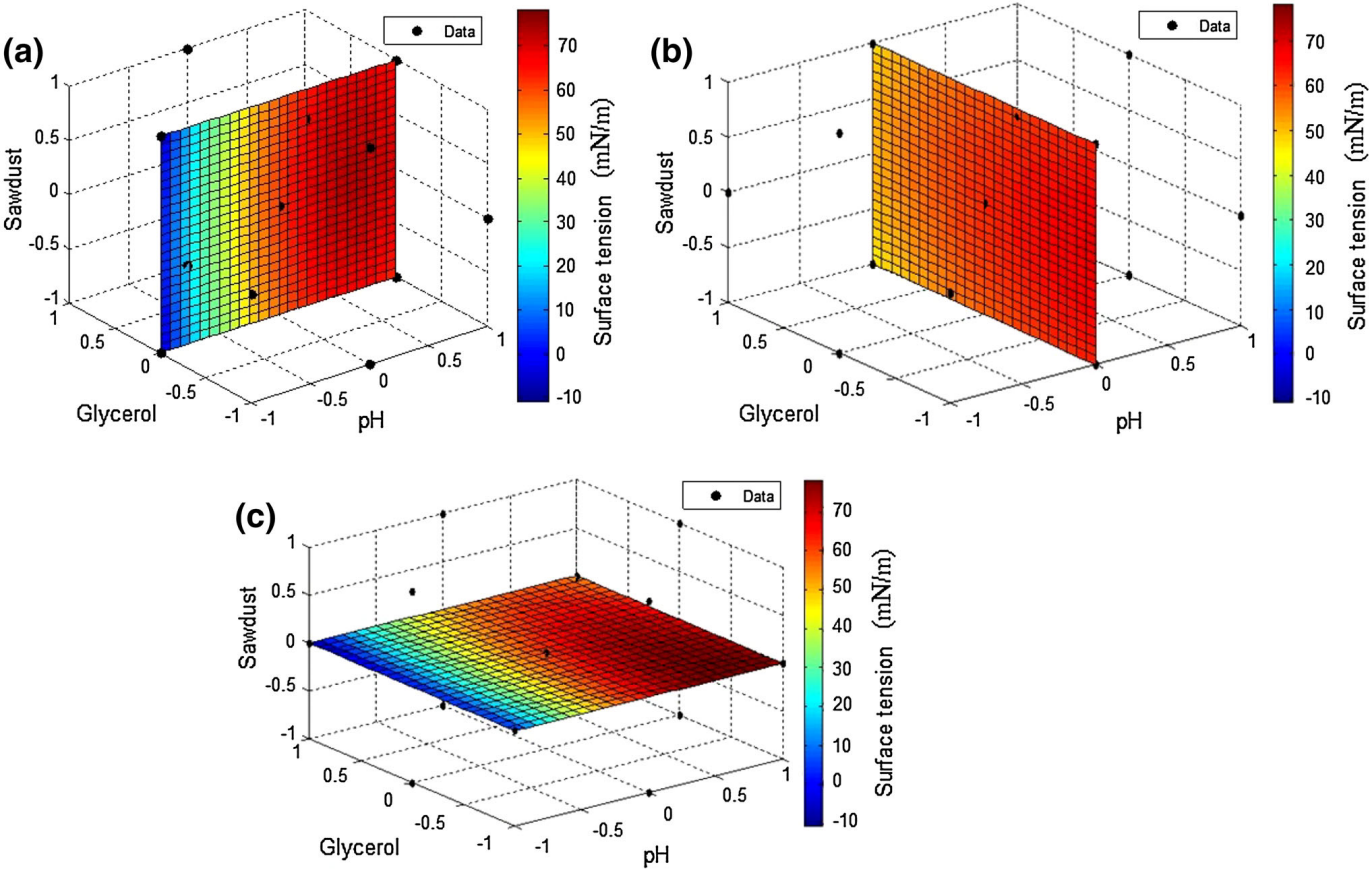
**a** Interaction of pH and sawdust while glycerol at its control value, **b** interaction of glycerol and sawdust while pH at its control value, **c** interaction of glycerol and pH while sawdust at its control value

In PBD approach, six parameters including carbon sources (groundnut oil and glycerol), renewable sources (groundnut husk and sawdust), and other factors (pH and inoculum level) were screened for biosurfactant production in 12 combinations with two test levels, and experiments were performed according to design matrix detailed in Table 1). From the PBD surface tension results, main effects were calculated at a confidence level of 95 % and are summarized in Table 2. Box–Behnken design (BBD) was adopted to optimize the levels of three identified substrates. The individual and interactive effects were noticed at three levels (−1, 0, and +1) of variables, and detailed design was given in Table 3. The response surface plot was represented as 3D plot with axes pH, sawdust, and glycerol at three levels of −1, 0, and 1 (Fig. 5). The contour plots depicted interactions of two variables with fixed value of variable at its control level (Fig. 6). The outcomes of Table 3 revealed that the factors *X*_1_, *X*_2_, and *X*_1_^2^ (*P < 0.05) were significant, but the remaining terms were not significant (Supplementary Table 3). Computed *F* value (22.89) and *P* < 0.001 was an indication of fitness of polynomial model (Table 4).

**Table 2:** Identification of significant substrates using PBD

Factor	Main effect	*t* stat
Sawdust	0.91	0.66
Groundnut husk	−0.91	−0.66
Glycerol	0.18	0.13
Groundnut oil	0.56	0.41
pH	−12.21	−8.86
Inoculum	−2.36	−1.71

**Table 3:** Three levels of substrates with actual and coded values and Box–Behnken experimental design matrix with experimental and predicted values of biosurfactant production

Trial	pH (*X*_1_)	Glycerol (*X*_2_) (ml)	Sawdust (*X*_3_) (g)	Surface tension (mN/m), experimental	Surface tension (mN/m), predicted
1	−1 (5)	−1 (1)	0 (7.5)	0	5.28
2	−1 (5)	+1 (3)	0 (7.5)	0	5.43
3	+1 (9)	−1 (1)	0 (7.5)	69.32	74.52
4	+1 (9)	+1 (3)	0 (7.5)	58.91	52.63
5	−1 (5)	0 (2)	−1 (5)	0	0.65
6	−1 (5)	0 (2)	+1 (10)	0	0.25
7	+1 (9)	0 (2)	−1 (5)	62.33	62.55
8	+1 (9)	0 (2)	+1 (10)	62.33	61.65
9	0 (7)	−1 (1)	−1 (5)	69.04	63.78
10	0 (7)	−1 (1)	+1 (10)	68.54	62.45
11	0 (7)	+1 (3)	−1 (5)	39.23	62.08
12	0 (7)	+1 (3)	+1 (10)	41.31	63.76
13	0 (7)	0 (2)	0 (7.5)	62.33	62.33
14	0 (7)	0 (2)	0 (7.5)	62.33	62.33
15	0 (7)	0 (2)	0 (7.5)	62.33	62.33

*R* ^2^ (adj) = 93.36 %; *R* ^2^ = 97.63 %

**Table 4:** Analysis of variance for the fitted second-order regression model for surface tension

Source	Df	SS	MS	*F*	*P* value
Regression	9	11,262.39	1,251.38	22.89	<0.001
Residual	5	273.39	54.68		
Total	14	11,535.78			

*Df* degrees of freedom, *SS* sum of squares, *MS* mean of squares, *F* Fischer’s test value, *P* value probability value

## Discussion

Selection of oil collapse and oil spreading methods was due to their strong advantages including simplicity, low cost, quick implementation, and use of relatively common equipment that is accessible in almost every microbiological laboratory. However, as expected, these methods are not perfect or flawless. The drop collapse method depends on the principle that a drop of liquid containing a biosurfactant collapses and spreads over the oily surface. There is a direct relationship between the diameter of the sample and concentration of the biosurfactant, and in contrast, the drop lacking biosurfactant remains beaded due to the hydrophobicity of the oil surface that cause aggregation of droplets (Youssef et al. 2004); Bodour and Miller-Maier 1998; Christofi and Ivshina 2002; Bodour et al. 2003; Tugrul and Cansunar 2005; Krepsky et al. 2007), but this method is not sensitive in detecting low levels of biosurfactant production. The emulsification index range of 7.8–63.3 % EA from biosurfactant was reported on kerosene (Techaosi et al. 2007). In *Rhodococcus* strain, emulsification activity of 63 % was reported using sunflower frying oil as substrate (Sadouk et al. 2008).

The *Pseudomonas aeruginosa* 2297 was extensively utilizes the renewable sources. During the growth of *Pseudomonas aeruginosa,* it produces surface-active compounds, which were measured as rhamnose equivalents (RE). Similar type of result was reported by *Pseudomonas* strain (Deziel et al. 1996). Biosurfactant production, like cell growth, depends on the availability of the substrate. Agro-industrial wastes are considered as the promising substrate for biosurfactant production and can alleviate many processing industrial waste management problems (Makkar et al. 2011). In the present TLC studies, we found that the *Pseudomonas aeruginosa* have shown two spots, which resembles the presence of two types of biosurfactants produced by used organisms, and similar results were observed by Koch et al. (1991) and Matsufuji et al. (1997) with *Pseudomonas aeruginosa*.

According to Sastoque-Cala et al. (2010), the specific rate of 0.109 h^−1^ was obtained by using MMS broth from *Pseudomonas fluorescence*. Regarding the production rate, the Leudeking and Piret model was used to describe this parameter, as it is versatile for fitting product formation data obtained from several fermentation processes (Bailey and Ollis 1986). According to the Rashedi et al. (2005), the maximum yield of rhamnolipid (*Y*_p/*x*_) from *Pseudomonas aeruginosa* was 0.21 g/g.

A suitable medium was also formulated through statistical optimization methodology since it has various advantages of being rapid and reliable in short listing of nutrients at varying concentrations leading to significant reduction in the total number of experiments. Two sequential steps of statistical approach such as Plackett–Burman design (PBD) and response surface methodology (RSM) were performed to design the optimized production medium for biosurfactant from *Pseudomonas* *aeruginosa*.

When additional factors are to be investigated, Plackett–Burman method may be adopted to find the variables influencing the metabolite production (Plackett and Burman 1944). This technique allows for the evaluation of ‘*n* − 1’ variables in ‘*n*’ experiments at high and low levels, and ‘*n*’ must be the multiple of 4. Each row in the design matrix represents one experimental run, and each column represents the high and low values of one factor. It requires that the frequency of each level of a variable in a given column should be equal. Once the data are obtained for each trial, statistical analysis can be performed to evaluate and rank factors by their degree of impact on the fermentation process. This ranking begins with the calculation of main effect and probability value of each factor. The main effect of the factor refers to the change in response over the entire range (−1 to +1).

For a variable *X*,

9 $${\text{Main effect}} = \frac{{\sum {X(H)} }}{{{\raise0.7ex\hbox{$n$} \!\mathord{\left/ {\vphantom {n 2}}\right.\kern-0pt} \!\lower0.7ex\hbox{$2$}}}} - \frac{{\sum {X(L)} }}{{{\raise0.7ex\hbox{$n$} \!\mathord{\left/ {\vphantom {n 2}}\right.\kern-0pt} \!\lower0.7ex\hbox{$2$}}}}$$

where Σ *X*(*H*): sum of high values of variable ‘*X*’ in one experimental run Σ *X*(*L*): sum of low values of variable ‘*X*’ in one experimental run *n*: number of experimental runs

 Software programs such as Microsoft Excel and MATLAB are used to calculate the main effects and *P* (probability) values of variables. A large estimate, either positive or negative, indicates that a factor has a large impact on metabolite productivity, while an estimate close to zero means that a factor has little or no effect. Generally, probability value <0.01 for a factor is considered as significant factor for the response of fermentation.

This statistical design is described by a first-order linear model as follows:

10 $$Y_{\text{S}} = \beta_{0} + \sum\limits_{i = 1}^{n} {\beta_{i} X_{i} }$$

*Y*_S_: surface tension, *β*_*i*_: linear coefficients, *X*_*i*_: factors.

The confidence level of components below 95 % in biosurfactant production was considered insignificant. Here, positive effect means reduction in surface tension, and negative effect means increase in surface tension. Hence, the effect for each component was considered as opposite from calculated values, i.e., lower surface tension means positive effect, and higher value means negative effect.

Of the tested variables, the positive effects on biosurfactant production have shown by sawdust, groundnut oil, and glycerol, while the negative impacts had given by pH, inoculums size, and groundnut husk. Initial pH of fermentation medium has got the highest impact on the surface tension. Significance of the present experimental could be validated through analysis of variance (*P* value <0.001). Proposed linear model was as follows:

11 $$Y_{\text{s}} = 56.841 + 0.908 \, X_{1} {-}0.908 \, X_{2} + 0.175 \, X_{3} + 0.558 \, X_{4} {-}12.802 \, X_{5} - 2.358X_{6}$$

Based on the main effects pH, sawdust and glycerol have profound effect on the biosurfactant production, and the exact optimal values of the individual factors are still unknown but can be optimized by RSM.

RSM is the process of adjusting variables toward the optimum response of fermentation. A second-order polynomial equation, fitted to data by multiple regression procedure, resulted in quadratic model, and it was given by Eq. 1.

1 $$Y = \beta_{0} + \, \beta_{1} X_{1} + \beta_{2} X_{2} + \beta_{3} X_{3} + \, \beta_{12} X_{1} X_{2} + \, \beta_{13} X_{1} X_{3} + \, \beta_{23} X_{2} X_{3} + \, \beta_{11} X_{1}^{2} + \beta_{22} X_{2}^{2} + \, \beta_{33} X_{3}^{2}$$

where *Y*––predicted response of fermentation *X*_1_, *X*_2_ and *X*_3_ are the coded settings for three factors *β*_0_––value of fitted response at the center point of the design *β*_1_, *β*_2_, and *β*_3_––linear coefficients *β*_12_, *β*_13_, and *β*_23_––interaction coefficients *β*_11_, *β*_22_, and *β*_33_––quadratic coefficients.

 An optimum combination of variables (pH: 7.37, sawdust: 7.656 g, and glycerol: 1.5 ml) was achieved through predicted plot of full quadratic model with surface tension of 70 ± 40 mN/m. To confirm the coefficients of second-order polynomial model by regression coefficient and analysis of variance (ANOVA), biosurfactant production was performed and the proposed model was written as follows:

12 $$Y = 62.3 \, + \, 31.6 \, X_{1} - \, 8.425 \, X_{2} + \, 0.2 \, X_{3} - 2.6 \, X_{1} X_{2} + \, 0.65 \, X_{2} X_{3} {-} \, 26.8 \, X_{1}^{2} {-} \, 3.45 \, X_{2}^{2} {-} \, 4.35 \, X_{3}^{2}$$

Results obtained from Table 3 illustrated that the factors *X*_1_, *X*_2_, and *X*_1_^2^ (*P < 0.05) were significant and the rest were not significant (Supplementary Table 3). Computed *F* value (22.89) and *P* < 0.001 was an indication of fitness of polynomial model (Table 4). The multiple correlation coefficient (*R* ^2^) was calculated as 97.63 %. This indicates that the second-order polynomial model could explain 97.63 % of variability in the response, and only 2.37 % of the total variations were not explained by the model. The adjusted *R* ^2^ (93.36 %) and predicted *R* ^2^ (97.63 %) were suggesting a high significance model used for analyzing the data.

Abalos et al. (2002) reported the utilization of response surface methodology to optimize the culture media for the production of rhamnolipids by *Pseudomonas aeruginosa* AT10. Similarly, Joshi et al. (2007) also reported the statistical optimization of medium components for the production of biosurfactant by *Bacillus licheniformis* K51. According to Joshi et al. (2007), Plackett–Burman and Box–Behnken designs are very effective statistical tools to improving biosurfactant production.

## Conclusion

In the present study, *P. aeruginosa* 2297 has showed good screening, emulsification, and surface-active properties. In the initial screening optimum process for carbon and renewable sources, sawdust, groundnut husk, groundnut, and glycerol were the best optimized substrates for biosurfactant production. The logistic equation and Leudeking–Piret models would fit to the growth of *Pseudomonas aeruginosa* on sawdust consumption and biosurfactant production. The statistical analysis of coefficient in Plackett–Burman design experiments demonstrates that pH, sawdust, and glycerol showed profound effect on the biosurfactant production. Optimization of these three selected variables while keeping the rest of the factors at their low levels through a Box–Behnken design shows maximum predicted biosurfactant production using pH: 7.37, sawdust: 7.656 g, and glycerol: 1.5 ml.

## Electronic supplementary material

Below is the link to the electronic supplementary material.

Supplementary material 1 (DOCX 14 kb)
