# Supplementary material for: Statistical approach to optimize production of biosurfactant by Pseudomonas aeruginosa 2297
Source: 3 Biotech. 2014 Mar 8;5(1):71–9. doi: 10.1007/s13205-014-0203-3 (PMC4327757; doi:10.1007/s13205-014-0203-3)
Supplement: Supplementary file 1 — Supplementary material 1 (DOCX 14 kb) [file 13205_2014_203_MOESM1_ESM.docx]

Table 1 ANOVA for growth of *Pseudomonas aeruginosa*

| *Source of Variation* | *SS* | *df* | *MS* | *F* | *P-value* |
| --- | --- | --- | --- | --- | --- |
| Rows | 1.72 | 5 | 0.34 | 11.14 | 0.01 |
| Columns | 0.03 | 1 | 0.033 | 1.09 | 0.34 |
| Error | 0.15 | 5 | 0.03 |  |  |
| Total | 1.91 | 11 |  |  |  |

*Df: Degrees of freedom; SS: Sum of squares; MS: Mean of squares;

*F: Fischer’s test value; P value: Probability value

Table 2 ANOVA for rhamnolipid production

| *Source of Variation* | *SS* | *df* | *MS* | *F* | *P-value* |
| --- | --- | --- | --- | --- | --- |
| Between Groups | 23641.87 | 2 | 11820.93 | 13.19 | 0.0002 |
| Within Groups | 16130.57 | 18 | 896.14 |  | <0.01 |
| Total | 39772.44 | 20 |  |  |  |

*Df: Degrees of freedom; SS: Sum of squares; MS: Mean of squares;

*F: Fischer’s test value; P value: Probability value

Table 3 Results of regression analysis of the second order polynomial model

|  |  | Coefficient | Standard error | t Stat | P value |
| --- | --- | --- | --- | --- | --- |
| Intercept | - | 62.31 | 4.27 | 14.59 | <0.0001**^*1^** |
| pH (X_1_) | Linear term | 31.62 | 2.61 | 12.09 | <0.0001**^*1^** |
| Glycerol (X2) | Linear term | -8.43 | 2.61 | -3.22 | 0.023**^*1^** |
| Sawdust (X3) | Linear term | 0.22 | 2.61 | 0.077 | 0.941^*2^ |
| X_1_X_2_ | Interaction term | -2.61 | 3.69 | -0.71 | 0.513 |
| X_1_X_3_ | Interaction term | Negligible | 3.69 | Negligible | 1.001^*2^ |
| X_2_X_3_ | Interaction term | 0.65 | 3.69 | 0.18 | 0.867^*2^ |
| X_1_^2^ | Quadratic term | -26.83 | 3.85 | -6.96 | 0.0009^*1^ |
| X_2_^2^ | Quadratic term | -3.45 | 3.85 | -0.89 | 0.411^*2^ |
| X_3_^2^ | Quadratic term | -4.35 | 3.85 | -1.13 | 0.309^*2^ |

**^*1^** Significant terms as p<0.05

**^*2^** NonSignificant terms as p>0.05
